# Tobacco smoking and the risk of Parkinson disease: A 65-year follow-up of 30,000 male British doctors

**DOI:** 10.1212/WNL.0000000000009437

**Published:** 2020-05-19

**Authors:** Benjamin Mappin-Kasirer, Hongchao Pan, Sarah Lewington, Jennifer Kizza, Richard Gray, Robert Clarke, Richard Peto

**Affiliations:** From the Clinical Trial Service Unit and Epidemiological Studies Unit (B.M.-K., H.P., S.L., J.K., R.G., R.C., R.P.), Nuffield Department of Population Health, University of Oxford; and Medical Research Council Population Health Research Unit (H.P., S.L.), Nuffield Department of Population Health, University of Oxford, United Kingdom.

## Abstract

**Objective:**

To investigate the causal relevance of current tobacco smoking for the risk of Parkinson disease (PD).

**Methods:**

We compared the risks of death from PD with smoking habits in 30,000 male doctors in the British Doctors cohort study in 1951 and in survivors who had been resurveyed periodically for 5 decades. Cause-specific mortality was monitored for 65 years and included 283 deaths from PD. The relative risks (RRs) of PD (and 95% confidence intervals [CIs]) were estimated using Cox models for smoking habits (smoking status, amount smoked, and years since quitting) at baseline or updated habits at resurvey.

**Results:**

The prevalence of current smoking declined progressively during follow-up from 67% to 8% between 1951 and 1998. The crude rates of PD death were lower in current smokers than in never smokers at baseline (30 vs 46/100,000 persons-years). After adjustment for age at risk, current smokers at baseline had a 30% lower risk of PD (RR 0.71; 95% CI 0.60–0.84), and continuing smokers classified using updated smoking habits at resurvey had a 40% lower risk (RR 0.60; 95% CI 0.46–0.77) of PD compared with never smokers. The risks of PD were inversely associated with the amount of tobacco smoked. The protective effect of current smoking vs never smoking for PD was attenuated by increasing duration since quitting smoking.

**Conclusions:**

In contrast to previous suggestions, the present report demonstrates a causally protective effect of current smoking on the risk of PD, which may provide insights into the etiology of PD.

Tobacco is a major cause of premature deaths in high-, middle-, and low-income countries, accounting for approximately 100 million deaths in the 20th century and a predicted 1 billion deaths in the 21st century.^1–6^ In the 50-year follow-up of male British doctors, current cigarette smoking caused more deaths from vascular, respiratory, and other neoplastic diseases than from lung cancer alone.^6–11^ The risks of almost all noncommunicable diseases (ischemic heart disease, cerebrovascular disease, diabetes, chronic lung disease, pneumonia, cirrhosis of the liver, and cancers of the mouth, esophagus, lung, and pancreas) were found to be higher in current smokers than in nonsmokers,^4–6^ except for Parkinson disease (PD), which is reported to be inversely associated with smoking.^11,12^

PD is a progressive neurodegenerative disorder characterized by clinical symptoms of bradykinesia, resting tremor, and muscular rigidity. Nonmotor symptoms of PD, including olfactory dysfunction, constipation, disordered sleep, and disordered mood, may precede the onset of motor symptoms by about a decade.^13–15^ The etiology of PD is poorly understood, but the pathology includes accumulation of Lewy bodies and loss of dopaminergic neurons in the substantia nigra region of the basal ganglia.^13,14^ PD is the most common movement disorder and the second most common neurodegenerative disease worldwide, affecting approximately 6.1 million people in 2015 and predicted to affect 9 million by 2030.^16,17^ Little is known about the modifiable risk factors for PD, but previous studies have reported positive associations of PD with head injury, pesticide exposure, and consumption of dairy products and inverse associations with caffeine, serum urate, physical activity, ibuprofen, and tobacco smoking.^18^

A meta-analysis of observational studies reported that current smoking was associated with a 60% lower risk of PD (relative risk [RR] 0.42; 95% confidence interval [CI] 0.38–0.47).^12^ However, there is substantial uncertainty about the causal relevance of this inverse association. A recent large case-control study, involving 1,808 PD cases and 1,876 controls in Denmark, suggested that the lower risk of PD in current smokers was an artifact of reverse causality bias, whereby early nonmotor signs of PD may include a reduced response to nicotine stimulation, prompting current smokers to quit smoking before the diagnosis of PD can be made.^19^ Most previous studies of smoking and the risk of PD have used a retrospective case-control study design (in which information on smoking habits was collected after the onset of disease), and such studies are particularly susceptible to the effects of reverse causality bias. Several prospective studies^11,12,20–22^ have also reported inverse associations of smoking with PD, but few such studies have included a sufficient number of PD cases or involved an adequate duration of follow-up to exclude effects of reverse causality bias. The aims of the present report, involving analyses of the 65-year follow-up of 30,000 male British doctors, were to assess the risks of PD associated with tobacco smoking habits, amount of tobacco smoked, and effects of duration since quitting smoking among ex-smokers.

## Methods

### Standard protocol approvals, registrations, and patient consents

No relevant research ethics committees existed in 1951, when the British Doctors Study was designed and baseline questionnaires were sent. Doctors who chose to answer the first questionnaire were informed by investigators of the implications and methods of the study. Participants could choose to withdraw at any stage during follow-up, and all data were kept confidential.

### Population

In 1951, all doctors who were registered with the British Medical Association and who were living in the United Kingdom were sent a postal questionnaire. Among 59,600 doctors contacted, two-thirds replied, and 34,439 (58.8%) male doctors provided complete data on their smoking habits. Surviving participants were resurveyed about changes in their smoking habits on 6 successive occasions between 1958 and 1998. Cause-specific mortality was monitored until November 30, 2016.

### Smoking habits

Information on smoking habits for each participant was collected using 7 self-completed postal questionnaires.^6–11^ Doctors classified themselves as current tobacco smokers, ex-smokers, or never smokers. Current smokers were further questioned about the age at which they started smoking, the amount of tobacco they smoked daily, and the type of tobacco smoked (cigarette or pipe). Ex-smokers were asked the same questions about the time at which they last smoked and were asked about quitting smoking. Never smokers were those doctors having never consumed as much as 1 cigarette per day, or the equivalent in pipe or cigar tobacco, for as long as 1 year. Resurvey questionnaires presented participants with their answers to the previous questionnaire and asked whether their habits had changed. Questionnaires were not sent to participants who had previously refused to answer questions, those who asked not to be contacted again, or those who had been struck off the medical register. After reminders, response rates to the 6 resurveys varied between 94% and 98%.

Cigarette, pipe, and cigar smokers were combined as tobacco smokers for all analyses. No exclusions were made on the basis of missing data. Nonresponders to a resurvey had their smoking habits carried forward from the most recent available survey. Tobacco smoking habits included classification of smoking status (current, ex-, and never smoker), average number of cigarettes per day, or the equivalent in pipe or cigar tobacco (hereafter referred to as “cigarette equivalents/day”), and time since last smoked defined prospectively from recruitment (smoke-free years before 1951 were not counted).

### Follow-up and PD

Information on vital status and cause of death for those who died was collected between November 1, 1951, and October 31, 2016, from national records, supplemented by personal inquiries. Cases were defined as doctors for whom death certificates indicated that PD was the underlying cause of death, *International Classification of Diseases, Seventh Revision (ICD-7)* code 350, *ICD-9* code 332.0, or *ICD-10* code G20 (hereafter referred to as “PD”). Doctors who withdrew before the end of the study or were lost to follow-up were included in analyses until withdrawal or attrition, after which they were censored. Complete follow-up of mortality was available for approximately 99% of participants in the study.

### Strategies to minimize reverse causality bias

The impact of reverse causality bias in observational studies can be minimized by ensuring that information about exposures is collected before the onset of the disease; excluding participants with previous disease at enrollment; and excluding a relevant period of early follow-up to minimize distortion of results by cases of disease that were undetected at enrollment. Hence, the first 10 years of follow-up were excluded from all analyses to minimize the effects of reverse causality bias.^23–26^

### Statistical methods

Two statistical models were used to investigate the associations of tobacco smoking with the risk of PD. First, a Cox proportional hazards model assessed the effect of smoking habits at baseline in 1951 (hereafter, the “baseline model”). Second, a Cox proportional hazards model assessed the effect of smoking habits updated periodically after each resurvey questionnaire (hereafter, the “updated model”). However, updates in smoking habits took effect in the model only 10 years after they were reported to ensure that changes in habits were not caused by underlying disease.

RRs of PD (RR, approximated by hazard ratios) were estimated for (1) current and ex-smokers relative to never smokers; (2) current smokers of 1–14 and 15 or more cigarette equivalents/day compared with never smokers; and (3) doctors not having smoked for 0–9 and 10 or more years compared with never smokers, after controlling for age at risk. RRs were adjusted for age at risk (i.e., current age during follow-up) rather than age at baseline to account for the increasing risk of PD associated with increasing age during the follow-up period. Group-specific CIs were calculated for RRs.^27,28^ Tests for trends in PD risk across categories of daily amount of tobacco smoked and time elapsed since cessation were conducted using likelihood ratio tests. Analyses were conducted using STATA 14.2 (StataCorp, USA), and figures were plotted using R 3.4.1 (R Foundation, Austria). All statistical tests were 2 sided, and statistical significance was defined as *p* < 0.05.

### Patient and public involvement

Participants were not involved in the design, conduct, analysis, or interpretation of the study. Results were disseminated by publications in medical journals and by the study website (ctsu.ox.ac.uk/research/british-doctors-study).

### Data availability

No additional data are available.

## Results

### Baseline characteristics of the study population

**Figure 1:**
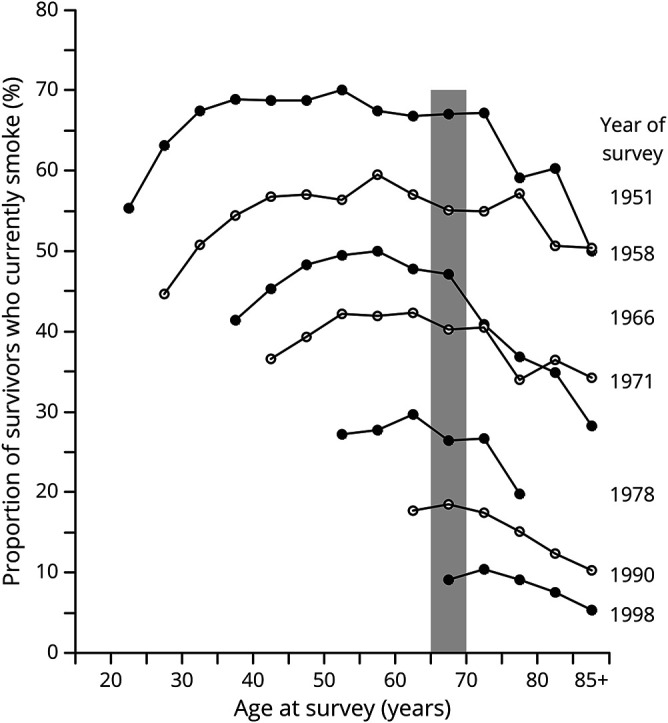
Smoking prevalence in surviving participants by age and year of survey Black and white points used only to distinguish between consecutive resurveys. The shaded bar shows the change of smoking prevalence from 1951 to 1998 in doctors aged 65–69 years.

Among 34,439 male doctors recruited in 1951, 467 (1.4%) requested no further questionnaires (mostly due to old age), 17 (0.05%) were excluded from the medical register (for professional misconduct), and follow-up was discontinued for 2,459 participants (7.1%) who were known to be alive but no longer living in the United Kingdom in 1971, leaving 29,737 (86.3%) male doctors for inclusion in the present analyses after exclusion of the first 10 years of follow-up (table). Figure 1 shows the decline in the prevalence of tobacco smoking in surviving doctors by age and survey year from 1951 to 1998. In doctors aged 65–69 years, the prevalence of current smoking declined from 67% in 1951 to 8% in 1998. The proportion of smokers who were cigarette smokers also declined from 63% in 1951 to 33% in 1998.

**Table:**
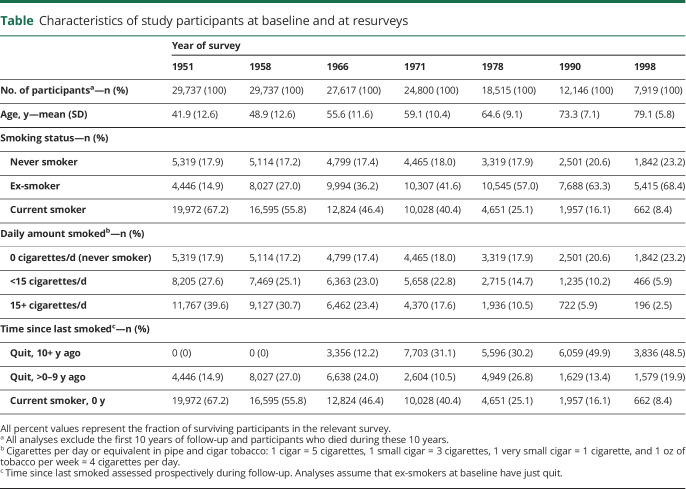
Characteristics of study participants at baseline and at resurveys

### Smoking status and the risk of PD

From 1951 to 2016, participants were followed on average for 35 years (range: 11–65 years). Excluding the first 10 years of follow-up, 25,379 deaths were reported during 743,920 person-years. Among these, 283 (1.1%) had PD listed as their underlying cause of death. After classification of smoking habits at baseline, the crude death rate from PD was lower in current vs nonsmokers (30 vs 46 per 100,000 person-years; unadjusted RR reduction of 34.7%). Doctors who died of PD were followed on average for 42 years and had a mean age at death of 82 years, whereas those who died of causes other than PD were followed on average for 35 years and had a mean age of death of 77 years.

**Figure 2:**
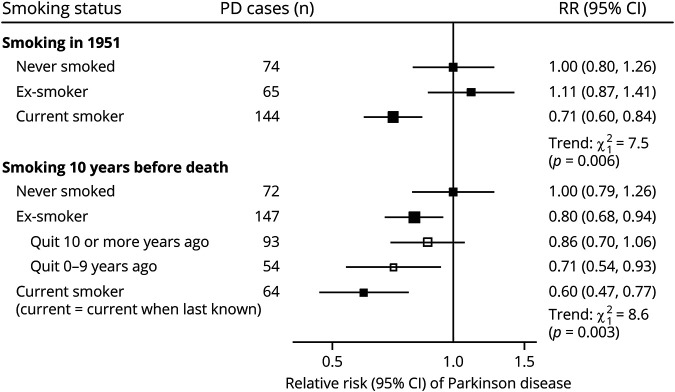
Relative risk of PD by smoking status (never, ex-, and current smoker) at baseline survey in 1951 and at resurveys and by years since quitting smoking The smoking status at resurveys was updated at each resurvey, with a 10-year time lag to minimize reverse causality bias. CI, group-specific confidence intervals. n, number of deaths from PD. Tests for trend of PD risk across categories of never, ex-, and current smoker. PD = Parkinson disease.

Current tobacco smoking was inversely associated with the risk of PD in both the baseline (*p* = 0.006) and updated (*p* = 0.003) models (figure 2). In the baseline model, doctors who reported current tobacco smoking in 1951 had about a 30% lower risk of PD compared with those who had never smoked on recruitment into the study (RR 0.71; 95% CI 0.60–0.84). In the updated model, current smokers had a 40% lower risk of PD compared with never smokers (RR 0.60; 95% CI 0.46–0.77).

### Mean daily amount smoked and the risk of PD

**Figure 3:**
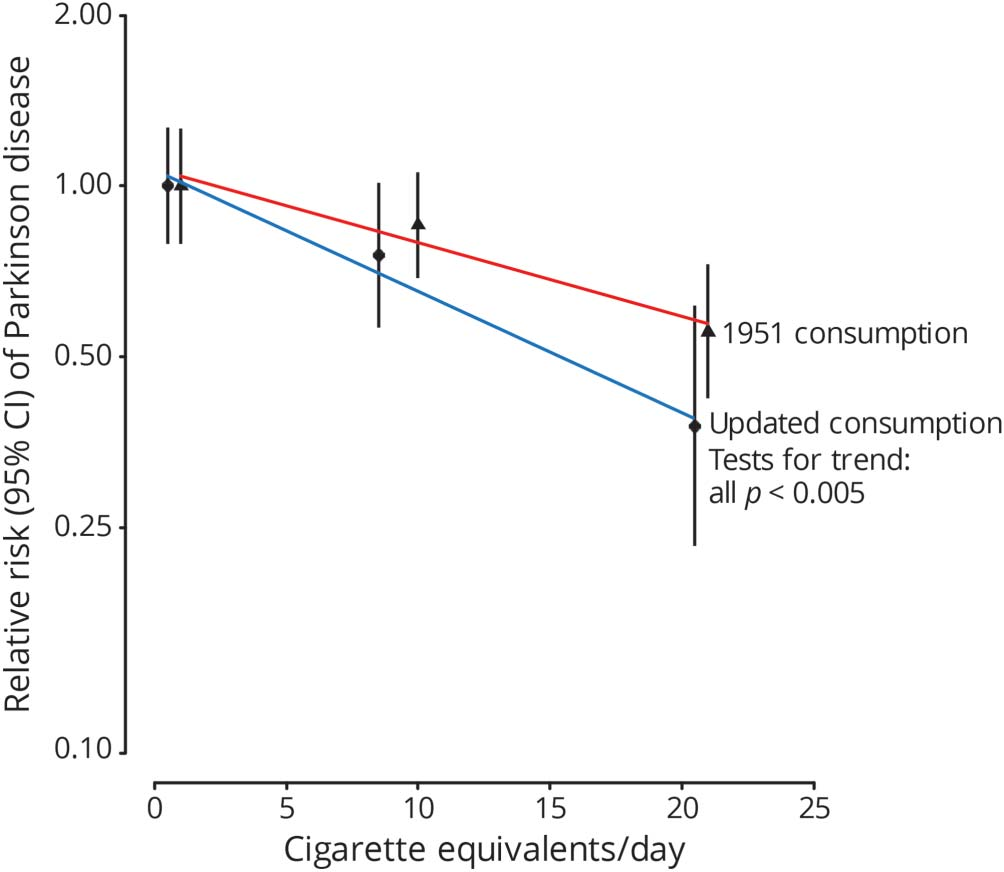
Association between daily amount of tobacco smoked and the risk of PD CI, group-specific confidence interval. The smoking status at resurveys was updated at each resurvey, with a 10-year time lag to minimize reverse causality bias. Cigarettes per day or equivalent in pipe and cigar tobacco: 1 cigar = 5 cigarettes, 1 small cigar = 3 cigarettes, 1 very small cigar = 1 cigarette, and 1 oz of tobacco per week = 4 cigarettes per day. Tests for trend in PD risk across categories of daily amount of tobacco smoked. Values are plotted at the daily amount smoked in each category, with an offset of 0.5 cigarette per day (updated consumption) or 1 cigarette/ per day (1951 consumption) to avoid overlap. The red and blue lines were the best fits across the data points. PD = Parkinson disease.

Analysis of the smoking habits of current smokers suggested that there was an inverse dose-response relationship between the daily amount of tobacco smoked and the risk of PD. Adjusting for age at risk, the association between daily amount smoked and PD risk was statistically significant in both the baseline (*p* = 0.0006) and updated (*p* = 0.002) models (figure 3).

### Duration since quitting smoking and the risk of PD

The risks of PD were estimated for ex- vs never smokers, and in the updated model, by the duration of time elapsed since quitting smoking (10 or more vs 0–9 years ago). Controlling for age at risk, duration since quitting was also associated with the risk of PD (figure 2). Compared with never smokers, those who quit smoking 10 or more years ago had a 14% lower risk of PD (RR 0.86; 95% CI 0.70–1.06), whereas those who quit 0–9 years ago had a 29% lower risk (RR 0.71; CI 0.54–0.93).

## Discussion

This study of 30,000 male British doctors, which minimized the risk of reverse causality by having very prolonged follow-up, demonstrated an inverse association of current tobacco smoking with the risk of PD. Compared with never smoking, current tobacco smoking was associated with a 30% lower risk of PD using smoking habits at baseline and with a 40% lower risk using smoking habits that were updated at sequential surveys. The risk of PD was inversely related to the amount smoked, and the protective effect of smoking on the risk of PD was attenuated with increasing duration of time since quitting smoking.

The chief strengths of this study include the prolonged duration of follow-up, serial resurveys of smoking habits in each decade over 5 decades, and the consistently high response rates to each survey. Furthermore, the present study compared the effects of smoking on PD using 2 statistical models that took account of changes in smoking habits, reverse causality, and age at risk of PD, each of which yielded consistent results.

A limitation of this report was that the analyses were based on only 283 PD cases. Participants included only male British doctors, and the brief survey questionnaires that yielded high response rates did not collect data on the family history of PD, caffeine intake, or other covariates. However, results of the present study are consistent with those from previous Western population–based cohorts involving both men and women that also included extensive multivariate adjustment. Nonetheless, studies correcting for reverse causality bias in non–Western populations, with adjustment for other major risk factors for PD, could confirm the generalizability of these findings.^12,18^ In addition, cases with PD as the associated (secondary) cause of death were not considered. It is possible that the association of tobacco smoking with PD as the associated cause of death might differ from that of smoking with PD as the underlying cause of death, particularly if concomitant disease in the former was related to smoking. However, the concordant results of the present report with those of previous prospective studies that included incident nonfatal PD cases suggest that any bias from relying on fatal cases where PD was the underlying cause of death is likely to be small. Furthermore, cases were defined using *ICD* codes for PD, which may not always account for distinction between primary PD and parkinsonism secondary to an unknown cause or between different clinical subtypes of PD. However, cases with PD as the underlying cause of death on death certificates were more likely to have more severe disease and more definite diagnoses of PD.^29^

The present analyses addressed concerns that the inverse associations observed between tobacco smoking and PD risk were an artifact of chance or bias. Even if the pathogenesis of PD begins 2 decades before the first motor manifestation of illness, smoking habits at baseline in 1951 would have been largely unaffected by premanifest disease, as they were recorded on average 42 years before death from PD and approximately 35 years before death from other causes. Hence, the protective effects of current smoking for PD are unlikely to be due to reverse causality bias. Because smoking is positively associated with many other causes of death, analyses that take account of competing risks would reduce the estimated death rate of PD to a greater extent in smokers than in nonsmokers. This, in turn, would tend to strengthen the inverse association of smoking with the risk of PD rather than dilute it.

The mechanisms underlying the inverse association between tobacco smoking and PD are not fully understood. Some studies have suggested that nicotine may have neuroprotective properties and stimulate the release of dopamine, but effects of other components of tobacco cannot be excluded.^30,31^ Recent studies have identified pathologic proteins in the nasal cavity and gut,^32,33^ and other studies have linked PD with derangements in lysosomal storage function.^34^ Given the effect of tobacco smoking on nasal and gut mucosa, it is possible that the inverse association of smoking with PD may be mediated by such mechanisms. Genome-wide association studies have identified genetic variants associated with PD risk^35,36^ and with particular smoking habits^37^ and provided some support for a causal relation between smoking habits and PD, albeit such studies have not yet elucidated the underlying mechanism.^38^

In all populations, the adverse effects of smoking on risks of vascular and respiratory diseases, neoplasms, and other noncommunicable diseases for which tobacco is the chief risk factor greatly exceed any protective effect of current tobacco smoking on the risk of PD. A trial of transdermal nicotine treatment to slow the progression of PD is currently underway (NCT01560754), but trials of primary prevention of PD are unlikely to be feasible. Nonetheless, the results of the present study suggest that efforts to characterize the biological mechanisms underlying the inverse association between tobacco and PD may be worthwhile and may contribute to a better etiologic understanding of PD.

## Glossary

CI: confidence interval
ICD: International Classification of Diseases
PD: Parkinson disease
RR: relative risk

## References

[1] Jha P, Peto R. Global effects of smoking, of quitting, and of taxing tobacco. N Engl J Med 2014;370:60–68. 24382066 10.1056/NEJMra1308383

[2] Jha P. Avoidable global cancer deaths and total deaths from smoking. Nat Rev Cancer 2009;9:655–664. 19693096 10.1038/nrc2703

[3] Peto R, Lopez A. The future worldwide health effects of current smoking patterns. In: Koop E, Pearson C, Schwarz M, editors. Critical Issues in Global Health. New York: Jossey-Bass; 2001:314–319.

[4] Chen Z, Peto R, Zhou M, et al. Contrasting male and female trends in tobacco-attributed mortality in China: evidence from successive nationwide prospective cohort studies. Lancet 2015;386:1447–1456. 26466050 10.1016/S0140-6736(15)00340-2 PMC4691901

[5] Pirie K, Peto R, Reeves GK, Green J, Beral V; Collaborators MWS. The 21st century hazards of smoking and benefits of stopping: a prospective study of one million women in the UK. Lancet 2013;381:133–141. 23107252 10.1016/S0140-6736(12)61720-6 PMC3547248

[6] Doll R, Peto R, Boreham J, Sutherland I. Mortality in relation to smoking: 50 years' observations on male British doctors. BMJ 2004;328:1519. 15213107 10.1136/bmj.38142.554479.AE PMC437139

[7] Doll R, Hill AB. The mortality of doctors in relation to their smoking habits. BMJ 1954;1:1451. 13160495 10.1136/bmj.1.4877.1451 PMC2085438

[8] Doll R, Hill AB. Lung cancer and other causes of death in relation to smoking. BMJ 1956;2:1071. 13364389 10.1136/bmj.2.5001.1071 PMC2035864

[9] Doll R, Hill AB. Mortality in relation to smoking: ten years' observations of British doctors. BMJ 1964;1:1399. 14135164 10.1136/bmj.1.5395.1399 PMC1814562

[10] Doll R, Peto R. Mortality in relation to smoking: 20 years' observations on male British doctors. BMJ 1976;2:1525–1536. 1009386 10.1136/bmj.2.6051.1525 PMC1690096

[11] Doll R, Peto R, Wheatley K, Gray R, Sutherland I. Mortality in relation to smoking: 40 years' observations on male British doctors. BMJ 1994;309:901–911. 7755693 10.1136/bmj.309.6959.901 PMC2541142

[12] Li X, Li W, Liu G, Shen X, Tang Y. Association between cigarette smoking and Parkinson's disease: a meta-analysis. Arch Gerontol Geriatr 2015;61:510–516. 26272284 10.1016/j.archger.2015.08.004

[13] Kalia LV, Lang AE. Parkinson's disease. Lancet 2015;386:896–912. 25904081 10.1016/S0140-6736(14)61393-3

[14] Postuma RB, Berg D, Stern M, et al. MDS clinical diagnostic criteria for Parkinson's disease. Mov Disord 2015;30:1591–1601. 26474316 10.1002/mds.26424

[15] Noyce AJ, Bestwick JP, Silveira-Moriyama L, et al. Meta-analysis of early nonmotor features and risk factors for Parkinson disease. Ann Neurol 2012;72:893–901. 23071076 10.1002/ana.23687 PMC3556649

[16] Vos T, Allen C, Arora M, et al. Global, regional, and national incidence, prevalence, and years lived with disability for 310 diseases and injuries, 1990–2015: a systematic analysis for the Global Burden of Disease Study 2015. Lancet 2016;388:1545. 27733282 10.1016/S0140-6736(16)31678-6 PMC5055577

[17] Dorsey E, Constantinescu R, Thompson J, et al. Projected number of people with Parkinson disease in the most populous nations, 2005 through 2030. Neurology 2007;68:384–386. 17082464 10.1212/01.wnl.0000247740.47667.03

[18] Ascherio A, Schwarzschild MA. The epidemiology of Parkinson's disease: risk factors and prevention. Lancet Neurol 2016;15:1257–1272. 27751556 10.1016/S1474-4422(16)30230-7

[19] Ritz B, Lee P-C, Lassen CF, Arah OA. Parkinson disease and smoking revisited ease of quitting is an early sign of the disease. Neurology 2014;83:1396–1402. 25217056 10.1212/WNL.0000000000000879 PMC4206154

[20] Rogot E, Murray JL. Smoking and causes of death among US veterans: 16 years of observation. Public Health Rep 1980;95:213. 7384406 PMC1422715

[21] Thacker E, O'reilly E, Weisskopf M, et al. Temporal relationship between cigarette smoking and risk of Parkinson disease. Neurology 2007;68:764–768. 17339584 10.1212/01.wnl.0000256374.50227.4b PMC2225169

[22] Chen H, Huang X, Guo X, et al. Smoking duration, intensity, and risk of Parkinson disease. Neurology 2010;74:878–884. 20220126 10.1212/WNL.0b013e3181d55f38 PMC2836869

[23] Hoehn MM, Yahr MD. Parkinsonism onset, progression, and mortality. Neurology 1967;17:427–442. 6067254 10.1212/wnl.17.5.427

[24] Di Rocco A, Molinari SP, Kollmeier B, Yahr MD. Parkinson's disease: progression and mortality in the L-DOPA era. Adv Neurol 1996;69:3–11. 8615142

[25] Hely MA, Morris JG, Reid WG, Trafficante R. Sydney multicenter study of Parkinson's disease: non-L-dopa–responsive problems dominate at 15 years. Mov Disord 2005;20:190–199. 15551331 10.1002/mds.20324

[26] Poewe W. The natural history of Parkinson's disease. J Neurol 2006;253:vii2–vii6. 17131223 10.1007/s00415-006-7002-7

[27] Easton DF, Peto J, Babiker AG. Floating absolute risk: an alternative to relative risk in survival and case-control analysis avoiding an arbitrary reference group. Stat Med 1991;10:1025–1035. 1652152 10.1002/sim.4780100703

[28] Plummer M. Improved estimates of floating absolute risk. Stat Med 2004;23:93–104. 14695642 10.1002/sim.1485

[29] Berg D, Lang AE, Postuma RB, et al. Changing the research criteria for the diagnosis of Parkinson's disease: obstacles and opportunities. Lancet Neurol 2013;12:514–524. 23582175 10.1016/S1474-4422(13)70047-4

[30] Baron JA. Cigarette smoking and Parkinson's disease. Neurology 1986;36:1490–1496. 3531917 10.1212/wnl.36.11.1490

[31] Quik M, Perez XA, Bordia T. Nicotine as a potential neuroprotective agent for Parkinson's disease. Mov Disord 2012;27:947–957. 22693036 10.1002/mds.25028 PMC3685410

[32] Braak H, Del Tredici K, Rüb U, De Vos RA, Steur ENJ, Braak E. Staging of brain pathology related to sporadic Parkinson's disease. Neurobiol Aging 2003;24:197–211. 12498954 10.1016/s0197-4580(02)00065-9

[33] Kim S, Kwon SH, Kam TI, et al. Transneuronal propagation of pathologic α-synuclein from the gut to the brain models Parkinson's disease. Neuron 2019;103:627–647.e7. 31255487 10.1016/j.neuron.2019.05.035 PMC6706297

[34] Klein AD, Mazzulli JR. Is Parkinson's disease a lysosomal disorder? Brain 2018;141:2255–2262. 29860491 10.1093/brain/awy147 PMC6061679

[35] Nalls MA, Pankratz N, Lill CM, et al. Large-scale meta-analysis of genome-wide association data identifies six new risk loci for Parkinson's disease. Nat Genet 2014;46:989–993. 25064009 10.1038/ng.3043 PMC4146673

[36] Lill CM. Genetics of Parkinson's disease. Mol Cell Probes 2016;30:386–396. 27818248 10.1016/j.mcp.2016.11.001

[37] Liu JZ, Tozzi F, Waterworth DM, et al. Meta-analysis and imputation refines the association of 15q25 with smoking quantity. Nat Genet 2010;42:436–440. 20418889 10.1038/ng.572 PMC3612983

[38] Grover S, Lill CM, Kasten M, Klein C, Del Greco F, Konig IR. Risky behaviours and Parkinson's disease. Neurology 2019;93:e1412–e1424. 31527283 10.1212/WNL.0000000000008245 PMC7010323

